# CircMCTP2 enhances the progression of bladder cancer by regulating the miR-99a-5p/FZD8 axis

**DOI:** 10.1186/s43046-024-00206-6

**Published:** 2024-03-18

**Authors:** Yan Liu, Kexin Zhang, Xianxu Yang

**Affiliations:** Department of Urinary Surgery, the First Affiliated Hospital of Jinzhou Medical University, Jinzhou, Liaoning Province 121000 China

**Keywords:** circMCTP2, miR-99a-5p, FZD8, Tumorigenesis

## Abstract

**Background:**

CircRNAs and miRNAs are involved in the progression of tumor. CircMCTP2 is considered as a novel tumor promoter. However, the exact functions of circMCTP2 in bladder cancer are still unclear. This study was designed to explore the underlying mechanisms of circMCTP2-modulated tumor development in bladder cancer.

**Methods:**

The present study is an original research. The levels of circMCTP2 in a total of 39 bladder cancer specimens and cell lines were determined by RT-qPCR. The expression of FZD8 in T24 and RT-4 cells treated with miR-99a-5p mimics were examined using western blotting. In addition, the proliferative, migrative and invasive abilities of transfected cells were determined by CCK8 and Transwell assays. Furthermore, the apoptosis of transfected cells was evaluated using flow cytometry. Dual luciferase reporter assay was performed to elucidate the relationship between miR-99a-5p and circMCTP2/FZD8.

**Results:**

The levels of circMCTP2 were elevated in bladder cancer samples and cells, and this was related to worse survival rate. Downregulation of circMCTP2 suppressed growth and metastasis of cells, whereas the apoptotic rate of cells was enhanced. The levels of miR-99a-5rp was elevated after the downregulation of circMCTP2. Moreover, reverse correlation between the expression of miR-99a-5p and circMCTP2 was revealed in bladder cancer specimens. Additionally, FZD8 was the putative target of miR-99a-5p and the mimics of miR-99a-5p inhibited the proliferation, migration and invasion of bladder cancer cells via the FZD8/Wnt-b-catenin axis. Moreover, circMCTP2 regulated the growth and metastasis of bladder cancer cells potentially through regulating the miR-99a-5p/FZD8/Wnt-b-catenin axis. In summary, circMCTP2 was considered as an oncogenic factor through regulating the miR-99a-5p/FZD8/Wnt-b-catenin axis.

**Conclusions:**

This novel signaling could regulate the biological behaviours of bladder cancer cells, and these findings highlighted circMCTP2 as a critical target for treating bladder cancer.

## Introduction

Bladder cancer is one of the main malignant tumors of the urinary system in China, with the highest incidence among male genitourinary tumors [1]. Factors such as smoking, unhealthy dietary habits and chemical exposure have led to an increasing trend in the incidence of this disease [2–4]. Although developments in treatment methods have maintained bladder cancer-related mortality at a stable level or even slightly reduced it in recent years, patients with genetic variations or downregulated gene transcription levels still have a poorer prognosis [5]. Thus, identifying potential biomarkers and possible pathogenic mechanisms associated with the prognosis of bladder cancer patients can provide a theoretical basis for the design of targeted drugs against bladder cancer and the development of personalized treatment plans.

CircRNAs are non-coding RNAs and they are broadly expressed however their detailed roles are still not fully understood [6]. Accumulating studies suggests that circRNAs are essential regulators of numerous biological behaviours [6–8]. CircRNAs are key factors which are associated with disease development, and they contribute to the initiation and development of tumor [8, 9]. For instance, circ_CLIP2 downregulate the levels of miR-195-5p by direct binding, then miR-195-5p further suppressed HMGB3 expression in glioma. [10]. In addition, circRNA-100338 is able to induce metastasis in hepatocellular carcinoma through enhancing invasiveness and angiogenesis [11]. Moreover, hsa_circ_0005986 is considered as a putative predictor of HCC progression and survival of patients with hepatocellular carcinoma [12]. Furthermore, cir-ITCH is a novel tumor suppressor that regulated the development of colorectal cancer [13]. Previous study has revealed that circMCTP2 facilitates the proliferation and metastasis of bladder carcinoma through modulating the miR-498/murine double minute-2 axis [14]. The detailed function of circMCTP2 in bladder cancer are still unclear.

MiRNAs are endogenous small non-coding RNA molecule that negatively regulates specific protein-encoding genes in a sequence-specific manner [15]. Bioinformatics analysis indicates that miRNAs can regulate 30–60% of protein-encoding genes in the human genome [16]. There is increasing evidence to suggest that abnormal expression of miRNA can act as oncogenes or tumor suppressors in various types of malignant tumors [17]. These miRNAs can disrupt tightly controlled RNA networks in cancer cells [17]. Therefore, identifying miRNAs with abnormal expression can provide important clues for studying the molecular mechanisms of the initiation, progression, and metastasis of bladder cancer. Previous studies have revealed that miR-99a-5p could function as tumor suppressor in bladder cancer via targeting the signaling pathways such mTOR and NOX4 [18–21].

FZD8 is a protein in human that is encoded by *FZD8* gene. It is located on cell membrane and is involved in the Wnt-b-catenin signaling pathway [22]. It is a receptor for Wnt proteins, and component of the Wnt-Fzd-LRP5-LRP6 complex that triggers beta-catenin signaling through inducing aggregation of receptor-ligand complexes into ribosome-sized signalosomes [23]. In this study, the expression profile and novel downstream molecules of circMCTP2 in bladder cancer was elucidated. Furthermore, the influences of circMCTP2 knockdown on the biological behavior alterations in bladder cancer cells were explored.

## Materials and methods

### Patient samples

The research protocol was reviewed by the Ethics Committee of the First Affiliated Hospital of Jinzhou Medical University (approval no.: 2020–436). Informed consents were received from all participants. 39 matched bladder cancer and para-carcinoma specimens were obtained from patients who underwent operation in the First Affiliated Hospital of Jinzhou Medical University (Jinzhou, China) from March 2019 to June 2021. No patients had been treated with pre-operative anti-tumor therapies. The age of the patients were ranged from 42 to 84 year-old. Additionally, metastasis was found in 21 participants. Then the samples were kept at -80 °C. The inclusion criteria were as followed: 1) Able to provide written in formed consent; 2) Documentary evidence of a pathologically confirmed primary bladder cancer; 3) Consent to access archival tumor material; 4) Ascertainment of demographic data, height and weight, drug history and family history of bladder cancer and other cancer. The exclusion criteria were as followed: 1) Inability to provide informed consent; 2) history of any other primary malignancy except bladder cancer; 3) patient underwent adjuvant treatment such as radio- and chemo-therapy.

### Cells

SV-HUC-1 and human bladder cancer cells (T24, 5637, J82 and RT4) were obtained from the Institute of Cell Biology (Shanghai, China). Cells were cultured using DMEM (Sigma, St. Louis, MO, USA) containing FBS (10%), penicillin (100 U/ml) and streptomycin (100 µg/ml; GE Healthcare Life Science) and incubated at 37℃ with 5% CO_2_.

### Transfections

Vectors that overexpress FZD8 (oe-FZD8) together with the control oe-NC, miR-99a-5p mimic together with the control miR-NC, and small interfering RNA targeting circMCTP2 (sh-circMCTP2) together with the control sh-NC were purchased from Gene Pharma (Shanghai, China). T24 and RT4 cells were inoculated and cultured using DMEM containing no antibiotics. ~ 25 nM of vectors were utilized for transfection. All the transfections were conducted using Lipofectamine 2000 (Thermo Fisher Scientific, Inc., Waltham, MA, USA). Twelve hour following transfection, culture media was replaced with DMEM with 10% FBS. Twenty-four hours following the transfection with sh-circMCTP2, cells were treated with GSK-3b inhibitor (SB216763, Enzo Life Sciences, Farmingdale, NY, USA) at a concentration of 10 µM. After twelve hours of treatment, cell viability, migration, invasion and apoptosis were evaluated.

### Rever transtription-quantitative polymerase chain reaction

TRIzol (Sobao Biotechnology, Shanghai, China) was utilized for the extraction of RNA from clinical specimens or T24 and RT4 cells. Briefly, cDNA was generated using PrimeScript™ RT (Invitrogen, Shanghai, China). Then, qPCR was conducted by ABI 7500 (Thermo Fisher Scientific, Inc.). The program used for qPCR were: 95℃ for 5 min, then 45 cycles of 95℃ for 15 s 60℃ for 30 s and 72˚C for 10 s. The expression of genes was evaluated using 2^−ΔΔCT^ method. The forward and reverse primers were: miR-99a-5p: forward, 5’-TGGCATAAACCCGTAGATCC-3’ and reverse, 5’-CCATAGAAGCGAGCTTGTG-3’; circMCTP2: forward, 5’- ACCAGAAGAGCCAGAGGAGTC-3’, and reverse, 5’-TGGCCTGGTCCGCTGTTTTAA-3’; FZD8: forward, 5’- GGACTACAACCGCACCGACCT-3’ and reverse, 5’- ACCACAGGCCGATCCAGAAGAC-3’; GAPDH: forward, 5’-CGCTCTCTG CTCCTCCTGTTC-3’ and reverse, 5’-ATCCGTTGACTCCGACCTTCAC-3’; U6: forward, 5’-GTAGATACTGCAGTACG-3’ and reverse, 5’-ATCGCATGACGTACCTGAGC-3’.

### Dual luciferase reporter assay

Dual luciferase reporter plasmids (circMCTP2-WT, circMCTP2-MUT, FZD8-WT, FZD8-MUT) were generated by Promega (Promega, USA). Liposome 2000 (Invitrogen, Arsbad, CA, USA) was utilized for co-treatment of 293 T cells with circMCTP2-WT, circMCTP2-MUT, FZD8-WT, FZD8-MUT and miR-99a-5p mimics or miR-NC. The activity of luciferase was examined 48 h following treatment.

### Western blotting

The lysis of T24 and RT4 cells was performed using RIPA buffer (Thermo Fisher Scientific). Then protein samples were separated by 10% SDS-PAGE and then transferred to PVDF membranes (EMD Millipore, Billerica, MA, USA). The membranes were further blocked using 5% skimmed milk for 1 h and incubated with primary antibodies against FZD8 (1:1000, cat. no. ab155093, Abcam, MA, USA), b-catenin (1:2000, cat. no. ab224803, Abcam), cyclin D1 (1:2000, cat. no. ab32053, Abcam) or GAPDH (1:2000, cat. no. ab9485, Abcam) in cold room overnight. The following day, membranes were incubated with HRP-conjugated secondary antibody (1:3000, cat. no. 7074, Cell Signaling Technology). The blots were visualized by ECL kit (Pierce Biotechnology; Thermo Fisher Scientific) and quantified using Quantity One software.

### CCK-8 assay

Briefly, T24 and RT4 cells were seeded on 96 wells plates. At 24, 48, 72 and 96 h post-treatment, 10 μl of CCK-8 solution (CCK-8; Dojindo Molecular Technologies, Inc., Japan) was aliquoted into each well. Then the cells were kept in the incubator for 2 h and the absorbance (wavelength = 450 nm) was recorded using microplate reader (Bio-Rad Laboratories, Inc., Hercules, CA, USA).

### Assessment of cell migration and invasion

Chambers precoated with Matrigel (pore size = 8 μm; ChenGong Biotechnology, Shanghai, China) was utilized to assess the invasive activity only. In brief, 5 × 10^4^ of treated cells were seeded onto the upper chamber, and 500ul of culture media was added to lower compartment. Twenty four hours later, cells left in the upper compartment were discarded. Then fixation of membrane was performed using 4% paraformaldehyde (10 min). Subsenquently, cells stained using crystal violet (0.5%) were counted using microscope.

### Evaluation of cell apoptosis

Apoptosis assay (Baiying Biotechnology, Guangzhou, China) was utilized to evaluate the apoptosis of T24 and RT4 cells. Cells were washed and well mixed with PBS. For the evaluation of apoptosis, cells were well suspended and staining with PI (10 µg/ml) and annexin V-FITC (50 µg/ml) was carried out. Cells were kept at 4˚C for half an hour in dark. Subsequently, the apoptotic rate of cells was determined using a cytometer (BD Biosciences, Franklin Lakes, NJ, USA), and the results were interpreted by CellQuest analysis software (BectonDickinson, USA).

### In vivo study

T24 cells transfected with sh-circMCTP2 or sh-NC were used for inoculation into nude mice. BALB/C nude mice were purchased from the Laboratory Animal Research Centre of Jinzhou Medical University. Briefly, the mice were housed under a temperature-controlled condition (22 ± 2˚C) with ~ 60% humidity, under a 12-h dark/light cycle with libitum access to food and water for at least three days before the experiments. Mice were randomly grouped (*n* = 5 in each group) and injected with T24 cells. A total of 1 × 10^7^ cells were diluted in 200 μl PBS and injected into the back subcutaneously. Mice were then monitored four times a week. 42 days following the injection, mice were sacrificed and the tumor tissues were evaluated. Tumor volume was calculated using the following formula: V (mm3) = (length x width2)/2.

### Statistical analysis

SPSS statistical software (version 17.0, SPSS Inc., Chicago, IL, USA) was utilized to analyze the data. Data were shown as mean ± standard error of mean (SEM). Difference between two comparison groups was interpreted using t-test, and that among more than two groups was interpreted by one-way ANOVA and post-hoc Tukey test. To evaluate the association of variables, Pearson’s correlation analysis was carried out. The survival rate was determined using Kaplan–Meier analysis, and log-rank method was conducted to investigate the difference of groups with high- and low-circMCTP2-expression. *P* < 0.05 was statistically significant.

## Results

### The levels of circMCTP2 were increased in bladder cancer samples and cell lines

**Fig. 1:**
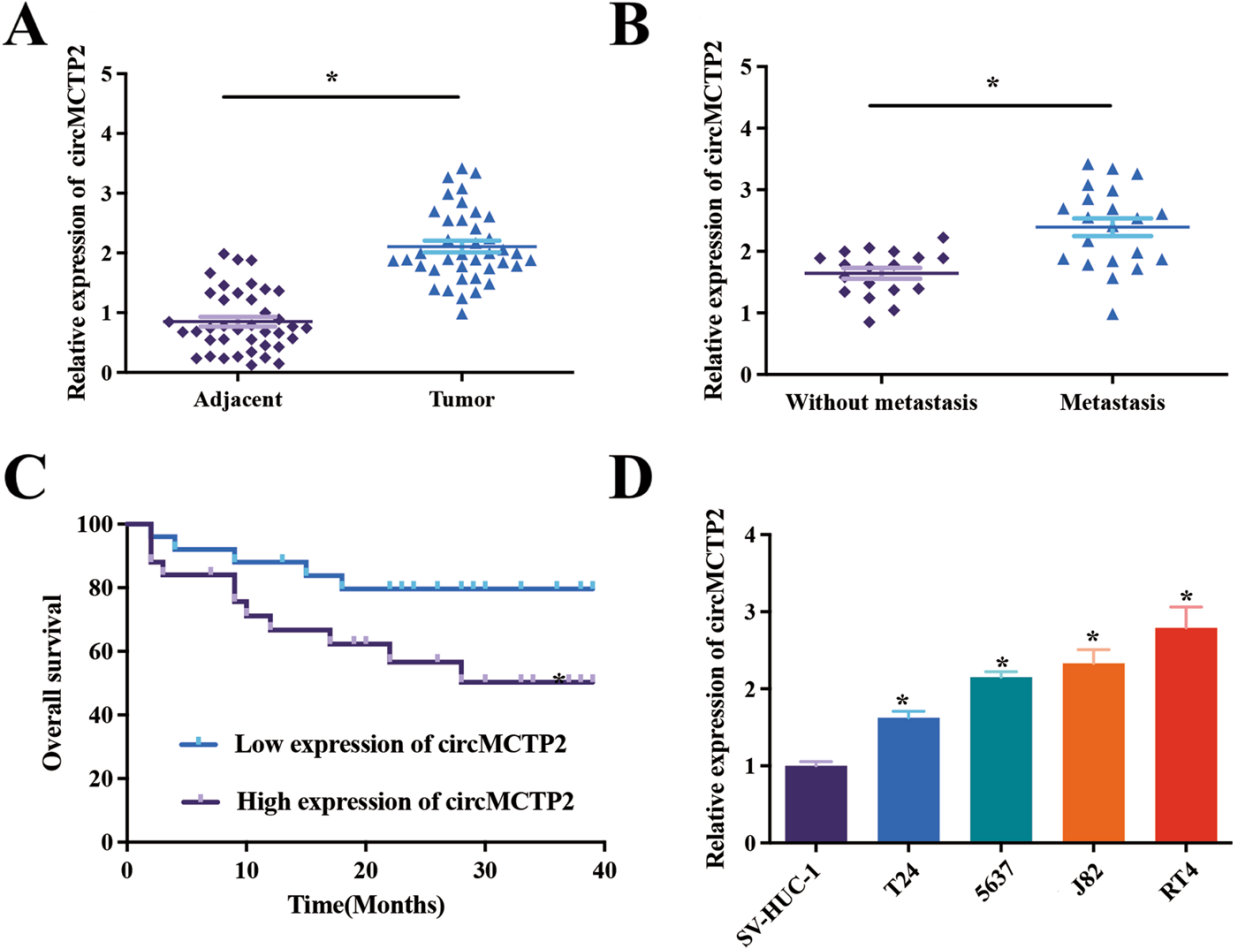
Up-regulation of circMCTP2 was revealed in bladder cancer samples and cell lines. **A** Up-regulation of circMCTP2 was revealed in bladder cancer specimens compared to paired non-carcinoma tissues. **B** Higher levels of circMCTP2 were observed in bladder cancer patients with metastasis. **C** Elevated circMCTP2 expression was related to poor prognosis in bladder cancer patients. **D** The levels of circMCTP2 were remarkably enhanced in bladder cancer cells. ^*^P < 0.05

The levels of circMCTP2 were notably upregulated in bladder cancer specimens than adjacent non-cancerous tissues (Fig. 1A). In addition, the expression of circMCTP2 in tissues from patients with/without metastasis was determined. The levels of circMCTP2 in individuals with metastasis were notably elevated compared to non-metastasis controls (Fig. 1B). Furthermore, higher expression of circMCTP2 was associated with worse overall survival in bladder cancer patients (Fig. 1C). Moreover, the levels of circMCTP2 in normal human uroepithelial SV-HUC-1and 5637, T24, RT4 and J82 cells was assessed using RT-qPCR. Our data revealed that the levels of circMCTP2 in bladder cancer cells were significantly upregulated (Fig. 1D). Additionally, T24 and RT4 cells were used for following experiments.

### Down-regulation of circMCTP2 inhibited the proliferation, migration and invasion of bladder cancer cells

**Fig. 2:**
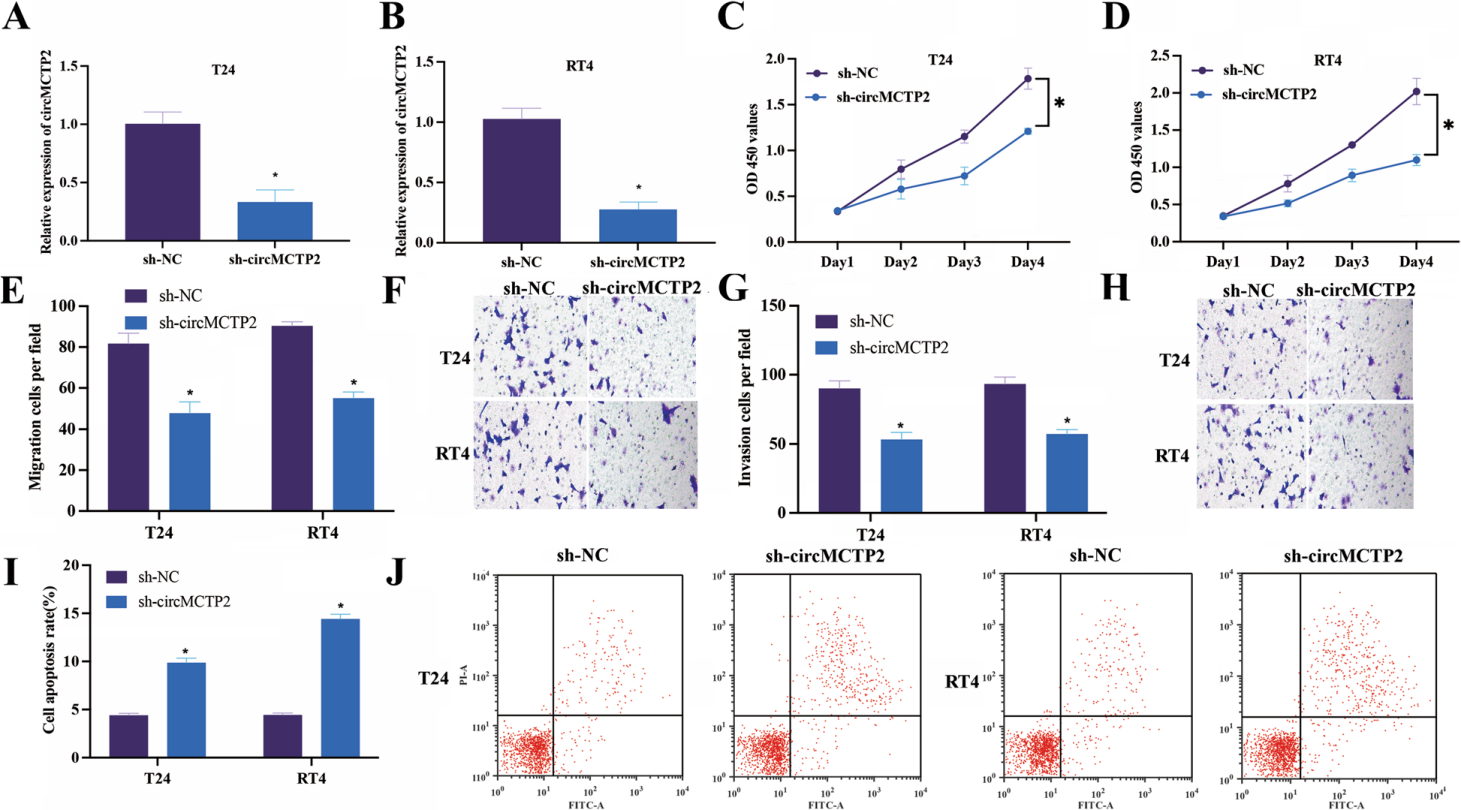
Down-regulation of circMCTP2 suppressed the proliferation, migration and invasion of bladder cancer cells. **A** and **B** Downregulation of circMCTP2 was observed in bladder cancer cells transfected with sh-circMCTP2. **C** and **D** The impacts of sh-circMCTP2 on the proliferative activity of T24 and RT4 cells were evaluated using CCK8 assay. **E**–**H** The effects of sh-circMCTP2 on the migrative and invasive abilities of bladder cancer cells were determined by Transwell assay. **I** and **J** The influences of circMCTP2 knockdown on the apoptosis of bladder cancer cells were examined using flow cytometry. ^*^P < 0.05

To elucidate the underlying regulatory roles of circMCTP2 in bladder cancer, T24 and RT4 cells were treated with sh-circMCTP2. The transfection efficiencies were confirmed by RT-qPCR (Fig. 2A and B). In addition, the proliferative, migrative and invasive abilities of cells were assessed after the treatment with sh-circMCTP2. The proliferation of cells treated with sh-circMCTP2 were notably suppressed (Fig. 2C and D). Furthermore, transfection with sh-circMCTP2 also remarkably inhibited cell migration (Fig. 2E and F) and invasion (Fig. 2G and H). In addition, the apoptosis of T24 and RT4 cells were notably enhanced following the treatment of sh-circMCTP2 (Fig. 2I and J).

### CircMCTP2 modulated the expression of miR-99a-5p

**Fig. 3:**
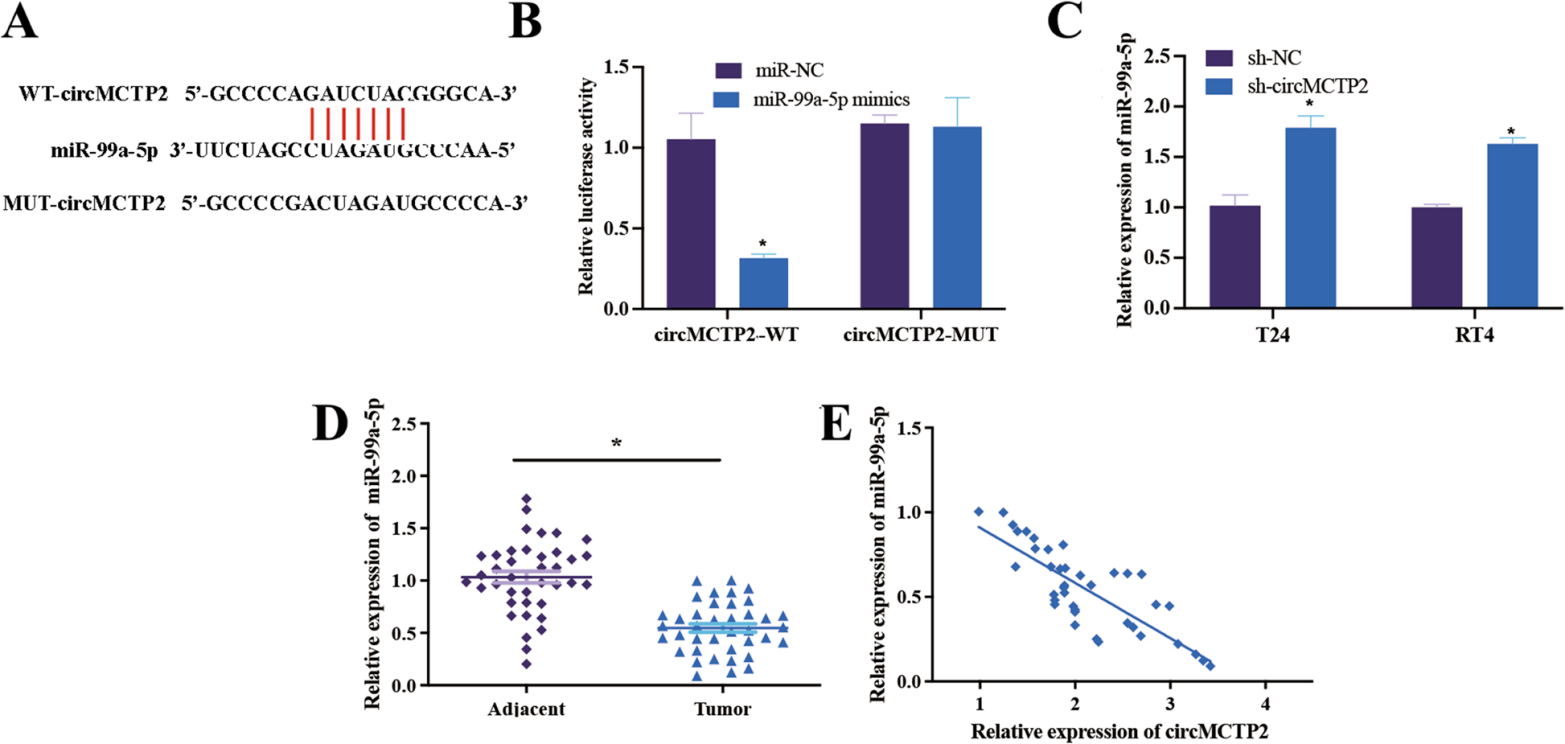
CircMCTP2 modulated the levels of miR-99a-5p. **A** The complementary binding fragments between miR-99a-5p and circMCTP2 were predicted. **B** Luciferase reporter assays were conducted to confirm the relationship between miR-99a-5p and circMCTP2. **C** The levels of miR-99a-5p was examined in T24 and RT-4 cells after the treatment with sh-circMCTP2. **D** The levels of miR-99a-5p were notably decreased in bladder cancer tissues. **I** Inverse correlation between miR-99a-5p and circMCTP2 expression was revealed in bladder cancer specimens. ^*^P < 0.05

Further function studies were conducted to explore the downstream molecules of circMCTP2. A binding fragment of miR-99a-5p to the 3’-UTR of circMCTP2 were presented in Fig. 3A. The results indicated that miR-99a-5p decreased the activity of the vectors containing wildtype 3’-UTR of circMCTP2 compared to the circMCTP2-MUT (Fig. 3B). Knockdown of circMCTP2 remarkably upregulated the expression of miR-99a-5p (Fig. 3C). Furthermore, the levels of miR-99a-5p were remarkably reduced in bladder cancer specimens than the para-carcinoma controls (Fig. 3D). In addition, reverse correlation between circMCTP2 and miR-99a-5p were detected in bladder cancer samples (Fig. 3E).

### FZD8 was the novel downstream molecule of miR-99a-5p

**Fig. 4:**
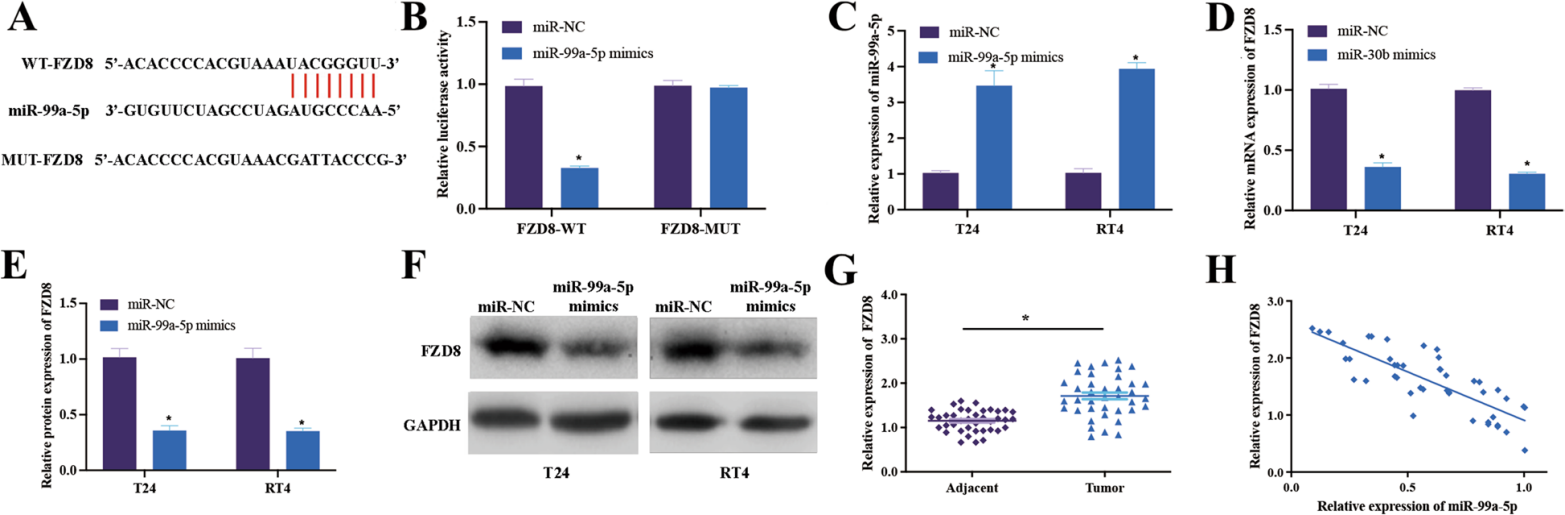
FZD8 was the novel downstream molecule of miR-99a-5p. **A** The complementary fragments between miR-99a-5p and the 3’-UTR of FZD8 were predicted. **B** Assay was conducted to evaluate the interaction between FZD8 and miR-99a-5p. **C** Upregulation of miR-99a-5p was detected after the treatment with miR-99q-5p mimics. **D**-**F** Both the mRNA and protein levels of FZD8 were examined in T24 and RT-4 cells following the treatment with miR-NC or miR-99a-5p mimics. **G** The expression levels of FZD8 were notably elevated in bladder cancer specimens. **H** The expression of miR-99a-5p and FZD8 were negatively correlated in bladder cancer samples. ^*^P < 0.05

Furhter studies were performed by TargetScan, and the results predicted that FZD8 contained putative binding fragments of miR-99a-5p. A binding sequence of miR-99a-5p the mRNA of FZD8 was shown in Fig. 4A. Moreover, our data suggested that miR-99a-5p decreased the luciferase activity of vectors carrying the WT 3’-UTR of FZD8 compared to the MUT control (Fig. 4B). Moreover, treatment with miR-99a-5p mimics remarkably elevated the levels of miR-99a-5p (Fig. 4C) and decreased the levels of FZD8, respectively (Fig. 4D-F). In addition, the levels of FZD8 were remarkably upregulated in bladder cancer specimens compared to the adjacent normal tissues (Fig. 4G). Furthermore, the levels of miR-99a-5p and FZD8 were reversely correlated in bladder cancer specimens (Fig. 4H). In summary, miR-99a-5p was able to suppress the expression FZD8.

### MiR-99a-5p affected the biological behaviours of bladder cancer cells via regulating the FZD8/Wnt-b-catenin axis

**Fig. 5:**
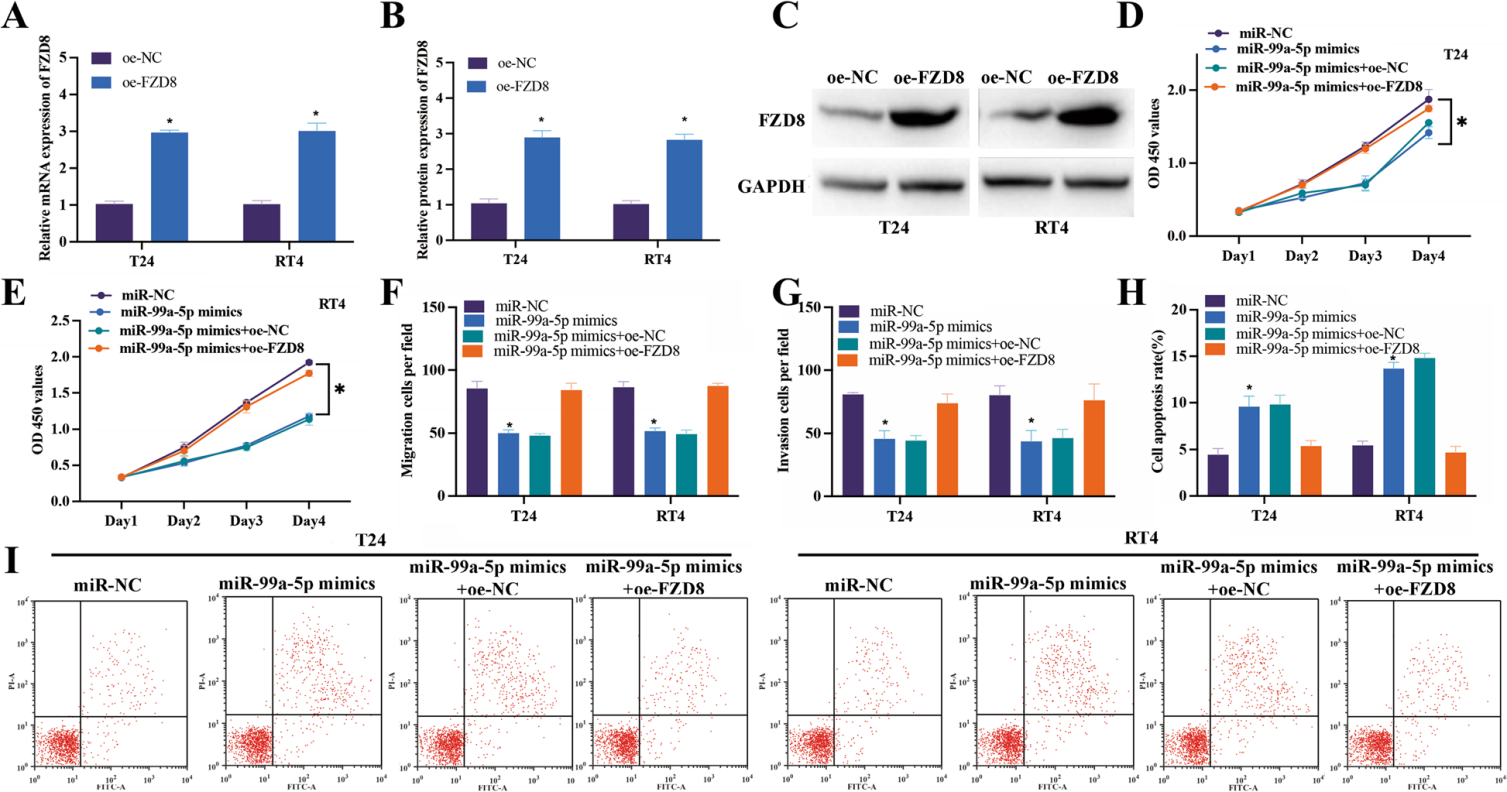
MiR-99a-5p suppressed the growth of bladder cancer cells through regulating the FZD8/Wnt-b-catenin axis. **A**-**C** Transfection with oe-FZD8 increased the expression of FZD8. **D** and **E** The influences of miR-99a-5p mimics and co-transfection with miR-99a-5p mimics and oe-FZD8 on the proliferation of T24 and RT-4 cells were examined. **F** and **G** The impacts of miR-99a-5p mimics and co-transfection with miR-99a-5p mimics and oe-FZD8 on migratory/invasive activities of bladder cancer cells were determined by Transwell method. **H** and **I** The apoptosis of T24 and RT-4 cells following the transfection with miR-99a-5p mimics or co-transfection with miR-99a-5p mimics and oe-FZD8 were elucidated. ^*^P < 0.05

**Fig. 6:**
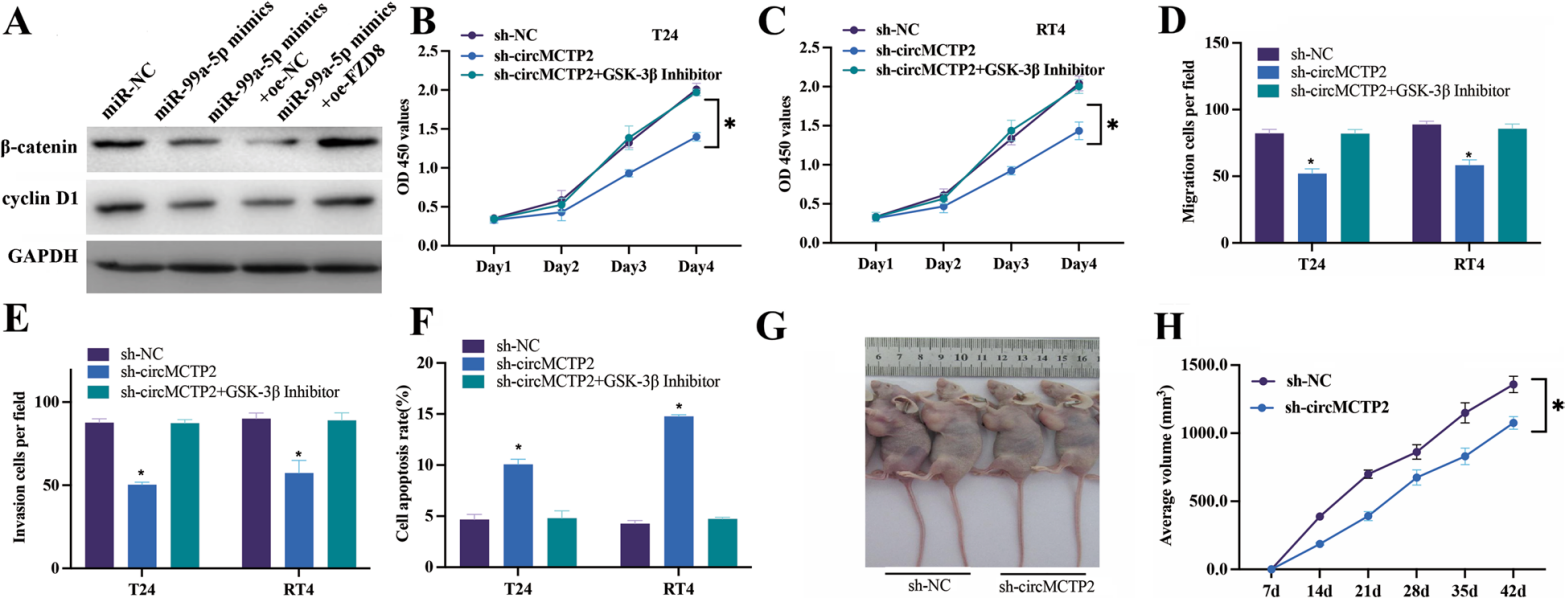
CircMCTP2 regulated the growth and metastasis of bladder cancer cells potentially via the miR-99a-5p/FZD8/Wnt-b-catenin axis. **A** The levels of both b-catenin and cyclin D1 were downregulated by the transfection with miR-99a-5p, which was abrogated by the treatment with oe-FZD8. **B**-**F** The inhibitory effects on the growth and metastasis of bladder cancer cells induced by sh-circMCTP2 were remarkably abolished by the treatment with GSK-3b inhibitor. **G** and **H** The growth of tumor was remarkably slowed in mice of sh-circMCTP2 group compared to sh-NC controls.^*^P < 0.05

In order to elucidate the impacts of the circMCTP2/miR-99a-5p/FZD8 axis on the biological behaviors of bladder cancer cells, T24 and RT4 cells were treated with miR-NC, miR-99a-5p mimics, or co-transfected with miR-99a-5p mimics and oe-NC/oe-FZD8. The results indicated that treatment with oe-FZD8 elevated the levels of FZD8 (Fig. 5A-C). Our data indicated that the proliferative (Fig. 5D and E), migrative (Fig. 5F) and invasive (Fig. 5G) activities of T24 and RT4 cells were remarkably downregulated by the treatment with miR-99a-5p mimics, while cell apoptotic rates (Fig. 5H and I) were notably promoted. Furthermore, the abovementioned biological behavior alterations were reversed by the treatment with oe-FZD8. Moreover, as the Wnt-b-catenin signaling is the novel downstream target of FZD8, the expression levels of Wnt-b-catenin-associated molecules were also determined. The levels of both b-catenin and cyclin D1 were downregulated by miR-99a-5p, which was abrogated by oe-FZD8 (Fig. 6A). Taken all together, miR-99a-5p was able to affect the growth of bladder cancer cells through regulating the FZD8/Wnt-b-catenin axis.

### CircMCTP2 regulate the growth and metastasis of bladder cancer cells potentially via the miR-99a-5p/FZD8/Wnt-b-catenin axis

In order to explore the downstream mechanisms of circMCTP2 in bladder cancer cells, T24 and RT4 cells were co-treated with sh-circMCTP2 and GSK-3b inhibitor, which activates the Wnt-b-catenin signaling. The inhibitory effects on the growth and metastasis of bladder cancer cells triggered by sh-circMCTP2 were notably abolished by the treatment with GSK-3b inhibitor (Fig. 6B-F). Moreover, the influences of sh-circMCTP2 on tumor growth were also evaluated in vivo. The average tumor volume was significantly reduced in mice of sh-circMCTP2 group compared to sh-NC controls (Fig. 6G and H).

## Discussion

Bladder cancer is a common malignant tumor, and the main approach to clinical treatment of bladder cancer at present is surgery combined with chemotherapy [1]. Due to the high toxicity and poor targeting of chemotherapy, its clinical application is limited, so there is a need to continue searching for new targeted and specific anti-bladder cancer drugs or biomarkers [3–5]. It has been reported that there is increasing evidence to suggest that abnormal expression of circRNAs and miRNAs in various types of malignant tumors can act as oncogenic factors or tumor suppressors. Moreover, identification of circRNAs and miRNAs with abnormal expression can provide important clues for studying the molecular mechanisms of the initiation, progression and metastasis of bladder cancer.

In our study, up-regulation of circMCTP2 was observed in bladder cancer specimens and cells, and this was related to poorer overall survival in bladder cancer patients. Moreover, downregulation of circMCTP2 suppressed the growth of bladder cancer cells. In consistence with these findings, previous study also revealed the up-regulation of circMCTP2 in bladder carcinoma, and circMCTP2 facilitates the proliferation and metastasis of bladder carcinoma through modulating the miR-498/murine double minute-2 axis [14]. These findings revealed the novel oncogenic role of circMCTP2, which was able to promote the growth and metastasis of bladder cancer cells. Furthermore, upregulation of circMCTP2 was also correlated with the occurrence of metastasis and poor overall survival rates in bladder cancer patients. Similarly, elevated expression of circMCTP2 was also observed in bladder cancer cells.

In addition, miR-99a-5p was identified as the putative target of circMCTP2, and circMCTP2 could downregulate the expression of miR-99a-5p. Furthermore, the expression levels of miR-99a-5p were downregulated in bladder cancer specimens, where the expression of circMCTP2 and miR-99a-5p were reversely correlated. Moreover, FZD8 was confirmed as the novel downstream molecule of miR-99a-5p, and miR-99a-5p reduced the mRNA and protein levels of FZD8. Additionally, the levels of FZD8 were increased in bladder cancer samples, where the expression of miR-99a-5p and FZD8 were negatively correlated. Moreover, miR-99a-5p suppressed the growth and metastasis of bladder cancer cells potentially via regulating the FZD8/Wnt-b-catenin axis, and these abovementioned inhibitory effects on the growth and metastasis of bladder cancer cells induced by sh-circMCTP2 were remarkably abolished by the treatment with GSK-3b inhibitor. In consistence with these findings, previous studies also revealed the potential roles of miR-99a-5p as a novel tumor suppressor [18–21]. It acts as tumor suppressor via targeting the mTOR and enhances RAD001-induced apoptosis in human urinary bladder urothelial carcinoma cells [18]. In addition, it was able to suppress cell proliferation, migration, and invasion by targeting isoprenylcysteine carboxylmethyltransferase in oral squamous cell carcinoma [19]. Furthermore, miR-99a-5p also inhibits glycolysis and induces cell apoptosis in cervical cancer by targeting RRAGD [20]. Moreover, miR-99a-5p regulates the proliferation, migration and invasion abilities of human oral carcinoma cells by targeting NOX4 [21]. In summary, miR-99a-5p could be a promising tumor suppressor in bladder cancer.

The potential roles of FZD8 in tumorigenesis have been previously reported. It promotes the cell proliferation and metastasis of renal cell carcinoma [24]. In addition, FZD8 indicates a poor prognosis and promotes gastric cancer invasion and metastasis via the b-catenin signaling pathway [25]. Moreover, FZD8 promotes bone metastasis in prostate cancer by activating canonical Wnt/β-catenin signaling [26]. In conclusion, our findings suggested the novel oncogenic role of FZD8 in bladder cancer.

Furthermore, the effects of sh-circMCTP2 on tumor growth were also examined in vivo, and the average tumor volume was significantly reduced in mice of sh-circMCTP2 group compared to sh-NC controls. Our results indicated that circMCTP2 regulated the growth and metastasis of bladder cancer cells potentially via the miR-99a-5p/FZD8/Wnt-b-catenin axis. However, there are some limitations in this study. For example, a total of 39 bladder specimens were examined and the sample size is relatively small, experiments on larger sample size should be conducted to confirm the existing findings. Compared to their linear compartments, circRNAs are resistance to RNAses and with long half-life period. Tumor metastasis is a complex process which involves various biomolecules. Identification of novel oncogenes is necessary. Due to marked ability of circMCTP2 to promote the development of bladder cancer cells, it could be possible to use an inhibitor to knockout circMCTP2 as a therapy to inhibit the progression of bladder cancer. Recently, RNA interference technologies have been developed and targeted treatment could be possible to lower the expression of circMCTP2 in bladder cancer cells. In addition, circMCTP2 could also be used as a novel biomarker to guide clinical diagnosis for bladder cancer patients.

## Conclusions

Taken all together, these data suggested the function of circMCTP2 on the development of tumor in bladder cancer. The levels of circMCTP2 were elevated in bladder cancer, and this promoted tumor progression via targeting the miR-99a-5p/FZD8/Wnt-b-catenin signaling. This putative pathway might be a critical target for treating bladder cancer.

## Data Availability

The datasets used and/or analyzed during the current study are available from the corresponding author on reasonable request.

## Abbreviations

circRNAs: circular RNAs
miRNAs: microRNAs
MUT: mutant
NC: negative control
WT: wildtype
UTR: untranslated region
